# A Physics-Informed Neural Network Scheme for Shortcuts to Adiabaticity in Three-Level Non-Hermitian Quantum Systems

**DOI:** 10.3390/e28080897

**Published:** 2026-08-10

**Authors:** Ming Liu, Fengxiao Huang, Siqi Zhang, Feng Yang, Wei Zhao, Junling Liu, Hong Li

**Affiliations:** 1 School of Data Science and Artificial Intelligence, Jilin Engineering Normal University, Changchun 130052, China; lium@jlenu.edu.cn; 2 Jilin Engineering Laboratory for Quantum Information Technology, Changchun 130052, China; zhangsq@jlenu.edu.cn (S.Z.); yangfeng@jlenu.edu.cn (F.Y.); zhaow168@nenu.edu.cn (W.Z.); 3 School of Civil Engineering, Changchun University of Architecture and Civil Engineering, Changchun 130607, China; huangfengxiao@jladi.edu.cn; 4 Institute for Interdisciplinary Quantum Information Technology, Jilin Engineering Normal University, Changchun 130052, China; 5 Institute of Quantum Science and Technology, Yanbian University, Yanji 133002, China

**Keywords:** shortcuts to adiabaticity, non-Hermitian quantum system, physics-informed neural network, fidelity

## Abstract

We propose a physics-informed neural network (PINN) scheme for designing shortcuts to adiabaticity in a three-level non-Hermitian quantum system. The PINN is used to solve an inverse control problem in which the state amplitudes and the auxiliary driving field are learned simultaneously from the Schrödinger residual, the initial and target population constraints and a probability conservation constraint on the control pulse. The learned compensation field counteracts the loss of the intermediate state and enables high-fidelity population inversion in an open-system setting. Importantly, the imposed probability conservation is treated as an auxiliary constraint along the learned trajectory rather than as an intrinsic property of the non-Hermitian Hamiltonian. Under this constrained evolution, the Hamiltonian expectation value evaluated on the obtained state remains real within numerical accuracy. Numerical simulations and independent propagation with the fitted control field verify the population inversion and exhibit strong generalization capability over a range of coupling strengths and dissipation rates, as verified by retraining the network independently for each parameter set. The results demonstrate that PINNs provide a flexible inverse design tool for non-Hermitian shortcut to adiabaticity protocols when the governing dynamics and physical constraints are explicitly incorporated into the loss function.

## 1. Introduction

Shortcuts to adiabaticity (STA) offer significant advantages in terms of both efficiency and reliability for quantum control [1,2]. To date, various STA methods have been developed, including transitionless quantum driving [3,4], invariant-based inverse engineering using Lewis–Riesenfeld invariants [5], and the fast-forward approach [6]. By constructing an effective Hamiltonian with a prescribed evolution path, these techniques enable quantum states to be rapidly transferred along a target trajectory while reproducing the desired adiabatic result. In recent years, non-Hermitian (NH) Hamiltonians have drawn considerable interest due to their distinctive spectral properties, including parity time symmetry and the presence of exceptional points. These systems show great promise in fields such as quantum optics, topological photonics, and sensing. Recently, research interest in shortcuts to adiabaticity has grown rapidly [7,8,9,10]. Non-Hermitian systems can exhibit highly degenerate points in their spectra, known as exceptional points (EPs), where both eigenvalues and their corresponding eigenmodes coalesce simultaneously [11,12,13]. Structures with EPs give rise to a variety of counterintuitive phenomena, including extreme sensitivity to perturbations [14], loss-induced transparency [15], and special topological properties [16]. Through a non-adiabatic compensation mechanism, the target state of a conventional adiabatic process can be accurately reproduced.

Compared to two-level systems, three-level systems offer richer energy-level structures and coupling channels. They provide a foundation for studying quantum phenomena such as interference effects, coherent population trapping, and more complex geometric phases. Research on shortcuts to adiabaticity in three-level non-Hermitian systems is of irreplaceable importance. Such systems are the simplest models that capture the intricate interference and coupling effects among multiple levels. Under non-Hermitian control, three-level systems exhibit novel dynamical behaviors, including evolution around higher-order exceptional points and chiral state transfer. This represents a key step toward a deeper understanding of the interplay between adiabaticity and dissipation in open quantum systems. The design of non-Hermitian shortcuts to adiabaticity requires solving differential equations, which has long been a research focus in computational mathematics. Many differential equations are difficult to solve using traditional analytical methods. Numerical methods for differential equations thus provide critical technical support for quantitative studies of complex systems. Deep learning, known for its powerful nonlinear approximation capability, has been widely applied to solving differential equations in various fields [17,18,19,20,21,22], offering new insights and approaches.

Physics-informed neural networks (PINNs) were first introduced by Raissi et al. in 2019 [23]. Their core idea is to embed governing equations, boundary conditions, and specific physical constraints directly into the loss function of a neural network, thereby training the network to find optimal solutions that satisfy physical laws [24,25,26,27,28,29,30,31,32,33,34,35]. In this approach, the solution to a differential equation is approximated by an artificial neural network. This provides a highly promising tool for addressing the design of shortcuts to adiabaticity in non-Hermitian three-level systems. By embedding physical information, the solution is ensured to comply with the fundamental principles of quantum mechanics and the peculiar properties of non-Hermitian systems [36,37,38,39,40,41], opening up broad prospects for scientific machine learning [42,43,44,45,46,47,48,49]. In collaboration with Cornell University, NTT Research scientist Logan Wright performed deep learning tasks using neural networks trained via backpropagation, leading to a significant reduction in the energy consumption of physical systems [50]. Chen and colleagues [51,52,53,54,55,56] investigated classical mathematical physics equations by applying PINNs to the sine-Gordon equation and the nonlinear Schrödinger equation, achieving favorable results. Chen Xi et al. [57] applied deep reinforcement learning to shortcuts to adiabaticity and designed high-precision control pulses. Raúl Coto et al. [58] used PINNs to solve the quantum master equation and obtained optimized control pulses. Very recently, PINNs have also been applied to population transfer in nuclear three-level systems, demonstrating their versatility beyond atomic and molecular systems [59].

The evolution of closed systems is generated by Hermitian Hamiltonians, ensuring norm conservation and unitarity. In open quantum systems and non-Hermitian quantum physics, however, non-Hermitian Hamiltonians are widely used to describe gain and loss, non-equilibrium dynamics, and exotic spectral structures. These Hamiltonians capture rich phenomena, including enhanced sensitivity near exceptional points and topological phase transitions, but their non-unitary evolution generally changes the norm of the state vector. A physically meaningful shortcut protocol must therefore specify how probability normalization, target transfer, and the compensating control field are enforced. In this work, we parameterize a time-dependent compensation term with a PINN and formulate the task as an inverse problem constrained by the non-Hermitian Schrödinger equation. The main contribution is not a proof that the Hamiltonian is pseudo-Hermitian; rather, it is a constrained inverse design procedure that learns a realizable auxiliary drive while keeping the obtained trajectory normalized and achieving the target population inversion. Within this constrained trajectory, the Hamiltonian expectation value is real within numerical accuracy, consistent with the condition obtained from differentiating the imposed norm constraint.

The structure of this paper is as follows. Section 2 introduces the PINN formulation and the three-level non-Hermitian STA model. Section 3 presents numerical simulations of the learned driving field, independent propagation verification, analytical fitting of the pulse, and robustness tests. Section 4 presents the conclusion.

## 2. Deep Learning Methods

### 2.1. PINNs Methods

**Figure 1:**
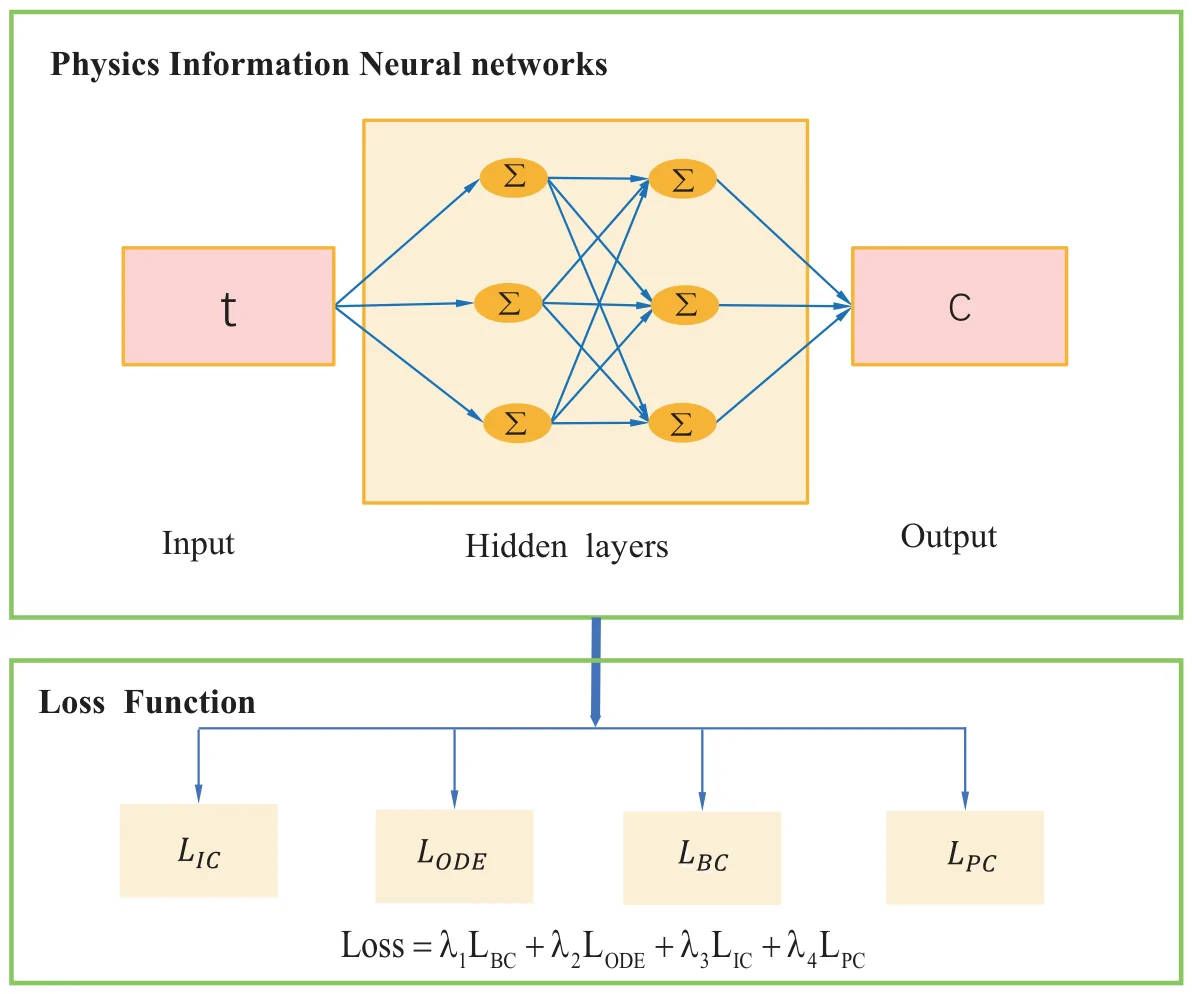
Neural network architecture.

A PINN is a deep learning model that incorporates physical laws into the training process and can be used as a numerical solver for differential equations. It is typically implemented using a fully connected backpropagation (BP) neural network. The model structure is shown in Figure 1. The neural network consists of interconnected neurons arranged in layers, including an input layer, hidden layers, and an output layer. Each layer contains a certain number of neurons. The input layer receives raw data. The hidden layers process the data layer by layer through weighting functions and activation functions. The output layer produces the final result. Such a neural network can approximate arbitrarily complex functions. The neural network can be described by the following function:

(1) $$y=u_{\theta }\left(x,\theta \right).$$

In the equation, *x* is the input data, *y* is the output data, and θ denotes the network parameters. Solving differential equations with a neural network requires training the network in advance. During training, the independent variables of the differential equation are fed into the neural network as input, and the corresponding outputs are generated. The derivatives of the network output at various orders are then computed and substituted into the differential equation. The differential equation, together with its initial and boundary conditions, is incorporated as constraints into the loss function. By iteratively adjusting the network parameters θ, the loss function value is gradually reduced until a convergence criterion is met or a preset number of training epochs is reached, yielding the trained neural network. The differential equation and its associated initial and boundary conditions are described as follows:

(2) $$F(u_{t}\left(t,x\right),u\left(t,x\right))=0\;t\in T,x\in \Omega ,$$

(3) B(u(t,x))=0t∈T,x∈Ω,

(4) I(u(t,x))=0t∈T,x∈Ω.

After training, the PINNs are capable of approximating the solution to the partial differential equation, as shown in the following expression

(5) $${\hat{u}}_{\theta }\left(t,x\right)\approx u\left(t,x\right).$$

Here, θ denotes the trainable parameters of the neural network. In a fully connected network, these parameters correspond to the weights *w* and biases *b* of each layer. The function ${\hat{u}}_{\theta }\left(t,x\right)$ is determined by the neural network with θ. Training the neural network is essentially a process of finding the optimal θ that minimizes the error between the model’s approximation and the true solution of the differential equation, i.e.,

(6) $$\theta^{*}=\arg\min_{\theta }L\left(u_{\theta }\right).$$

For a general PINN, the loss function is constructed from the equation residual and the constraints that define the problem. In the present inverse control setting, the loss contains the dynamical residual, the initial condition, the target final population and the probability conservation constraint: The loss function of a PINNs consists of four main components: the partial differential equation residual loss term $L_{\mathrm{ODE}}$, the boundary condition loss term $L_{\mathrm{BC}}$, the initial condition loss term $L_{\mathrm{IC}}$, and the probability conservation term $L_{\mathrm{PC}}$. The loss function is defined as follows:

(7) $$\mathrm{Loss}=\lambda_{1}L_{BC}+\lambda_{2}L_{ODE}+\lambda_{3}L_{IC}+\lambda_{4}L_{PC},$$

where

(8) $$L_{BC}=\frac{1}{N}\sum_{i=1}^{N}{\left|{\hat{u}}^{i}\left(t_{x},x_{B}\right)-u^{i}\left(t_{x},x_{B}\right)\right|}^{2},$$

(9) $$L_{ODE}=\frac{1}{N}\sum_{i=1}^{N}{\left|F({\hat{u}}_{t}^{i}\left(t,x\right),{\hat{u}}^{i}\left(t,x\right))\right|}^{2},$$

(10) $$L_{IC}=\frac{1}{N}\sum_{i=1}^{N}{\left|{\hat{u}}^{i}\left(t_{I},x\right)-u^{i}\left(t_{I},x\right)\right|}^{2},$$

(11) $$L_{PC}=\frac{1}{N}\sum_{i=1}^{N}{\left|{∥{\hat{u}}^{i}\left(t,x\right)∥}^{2}-1\right|}^{2}.$$

In the equation, $u^{i}\left(t_{I},x\right)$ and $u^{i}\left(t,x_{B}\right)$ denote the initial condition and boundary condition of the differential equation about analytical solution. ${\hat{u}}^{i}\left(t_{I},x\right)$ and ${\hat{u}}^{i}\left(t,x_{B}\right)$ denote the initial condition and boundary condition of the differential equation about numerical solution, respectively. $\lambda_{i}$ is the weight of the loss term, which during training prevents any single loss term from becoming too large or too small, thereby allowing the other terms to continue decreasing.

### 2.2. STAPINNs Method

In a three-level quantum system, the evolution satisfies the Schrödinger equation.

(12) $$i\hbar \partial_{t}\vec{\psi }\left(t\right)=H\left(t\right)\vec{\psi }\left(t\right),$$

where $\vec{\psi }\left(t\right)={\left[c_{1}\left(t\right),c_{2}\left(t\right),c_{3}\left(t\right)\right]}^{T}$ is the amplitude of vector containing the states |1〉 and |3〉 along with the intermediate state |2〉 of the three-level system, and the time-dependent Hamiltonian under the rotating-wave approximation is expressed as

(13) $$H_{0}\left(t\right)=\frac{\hbar }{2}\left(\begin{array}{ccc} 0 & \Omega_{P}\left(t\right) & 0\\ \Omega_{P}\left(t\right) & \Delta -i\Gamma & \Omega_{S}\left(t\right)\\ 0 & \Omega_{S}\left(t\right) & 0 \end{array}\right),$$

where $\Omega_{P}\left(t\right)$ and $\Omega_{S}\left(t\right)$ are the Rabi frequencies of the external optical field P and S pulses, respectively, Δ(t) is the detuning of the pump field, and Γ is the decay rate of the intermediate state |2〉. The population of the ground state |1〉 is initially set to unity, while those of |2〉 and |3〉 are initially zero; the time-dependent population transfer among the three states reflects the energy transfer process among them. In this paper, a PINN is used to optimize the auxiliary driving term for shortcuts to adiabaticity. The supplementary Hamiltonian takes the form:

(14) $$H_{1}\left(t\right)=\frac{\hbar }{2}\left(\begin{array}{ccc} 0 & 0 & i\Omega_{a}\left(t\right)\\ 0 & 0 & 0\\ -i\Omega_{a}\left(t\right) & 0 & 0 \end{array}\right),$$

where the auxiliary driving field function $\Omega_{a}\left(t\right)$ is purely real. The total Hamiltonian takes the following form:

(15) $$H\left(t\right)=H_{0}\left(t\right)+H_{1}\left(t\right),$$

H(t) satisfies the Schrödinger equation.

(16) $$i\hbar \dot{\vec{c}}\left(t\right)=H\left(t\right)\vec{c}\left(t\right)=\frac{\hbar }{2}\left(\begin{array}{ccc} 0 & \Omega_{P}\left(t\right) & i\Omega_{a}\left(t\right)\\ \Omega_{P}\left(t\right) & \Delta -i\Gamma & \Omega_{S}\left(t\right)\\ -i\Omega_{a}\left(t\right) & \Omega_{S}\left(t\right) & 0 \end{array}\right)\left(\begin{array}{c} c_{1}\\ c_{2}\\ c_{3} \end{array}\right).$$

The equation can be expanded into three time-dependent differential equations:

(17) $$\left\{\begin{array}{c} 2i{\dot{c}}_{1}=\Omega_{P}\left(t\right)c_{2}+i\Omega_{a}\left(t\right)c_{3}\\ 2i{\dot{c}}_{2}=\Omega_{P}\left(t\right)c_{1}+\left[\Delta \left(t\right)-i\Gamma \right]c_{2}+\Omega_{S}\left(t\right)c_{3}\\ 2i{\dot{c}}_{3}=-i\Omega_{a}\left(t\right)c_{1}+\Omega_{S}\left(t\right)c_{2} \end{array}\right..$$

In these equations, the term $i\Omega_{a}$ is the auxiliary imaginary coupling between states |1〉 and |3〉, $\vec{c}\left(t\right)$ is the solution to the equation, which is a function of time *t*. The shortcuts-to-adiabaticity method achieves an adiabatic process through driving control. Using the PINN model, the goal is to find $\Omega_{a}\left(t\right)$ that satisfies the dynamical equation and the imposed physical constraints. This problem is transformed into solving a parameterized differential equation. A BP neural network is used to approximate the solution to the differential equation. The quantities $\vec{c}$ and $\Omega_{a}$ are taken as the outputs of the neural network, with *t* as the input. The neural network can be abstractly represented as the following function:

(18) $$\left(\vec{c},\Omega_{a}\right)=N\left(t\right).$$

In the equation, $\vec{c}={\left(c_{1},c_{2},c_{3}\right)}^{T}$, $\Omega_{a}\left(t\right)$ is the driving term and serves as a time-dependent parameter of the differential equation. Both $\vec{c}$ and $\Omega_{a}\left(t\right)$ are outputs of the neural network. A PINN approximates the solution N(t) of the differential equation by minimizing the loss function of the neural network. The driving functions $\Omega_{a}\left(t\right)$ under this solution are also obtained. This neural network is referred to as Shortcuts to Adiabaticity PINNs (STAPINNs).

### 2.3. Design of STAPINNs

Figure 1 shows the schematic diagram of the PINN used in this work. The neural network architecture is as follows. A fully connected BP neural network with a layered structure is adopted. The layers are of three types: input layer, output layer, and hidden layers. In this work, the neural network consists of eight hidden layers, one input layer, and one output layer. The input layer contains a single node, which takes time *t* as the input value. The output layer has seven output nodes. The state functions and driving functions from the differential equations serve as the output values. For a three-level system, there are three states. Since the states are complex-valued, each state is split into its real and imaginary parts, resulting in six output nodes for the state components. The real-valued control amplitude $\Omega_{a}\left(t\right)$, which enters the Hamiltonian through the imaginary coupling $i\Omega_{a}\left(t\right)$, requires one additional output node. Each hidden layer contains 100 nodes. The objective is to optimize $\Omega_{a}\left(t\right)$ subject to the dynamical equation, the initial state ${\left(1,0,0\right)}^{T}$, the target final population $|c_{3}\left(t_{f}\right){|}^{2}≃1$, the norm constraint, and the pulse regularization terms described above. The value of time *t* is on the order of $1\times 10^{-10}\;s$, and the coefficients in the differential equation are also relatively large. This makes them unsuitable for direct use as inputs to the neural network. Therefore, the data used for training need to be regularized and nondimensionalized. In this work, the Allen–Eberly (AE) model is adopted, which takes the following form:

(19) $$\Omega_{P}=\Omega_{0}e^{\frac{-{\left(t-t_{f}/2-\tau \right)}^{2}}{\sigma^{2}}},$$

(20) $$\Omega_{S}=\Omega_{0}e^{\frac{-{\left(t-t_{f}/2+\tau \right)}^{2}}{\sigma^{2}}}.$$

where $\Omega_{0}$, τ and σ are parameters, and *t* denotes the system evolution time, $t_{f}$ is the final evolution time. The algorithm is implemented in Python 3.7, and the artificial neural network model is defined using PyTorch v2.13.0. Automatic differentiation is achieved through torch. The MSELoss function from PyTorch is used to evaluate each squared residual in L. Automatic differentiation is used to compute both ${\dot{\psi }}_{\theta }\left(t\right)$ and, when regularization is applied, ${\dot{\Omega }}_{a,\theta }\left(t\right)$.

During training, the input value is time *t*, and the sample points *t* are obtained from sampling results. The number of sampling points is N=50. At each training epoch, these 50 sample values are fed in batch into the input layer nodes. The output values consist of the population $c_{i}$ and the driving term $\Omega_{a}\left(t\right)$ during the system evolution. The previously defined loss function is used to train the neural network. Once trained, the network can approximate the solution to the differential equation and simulate the adiabatic evolution process.

## 3. Numerical Simulation

**Figure 2:**
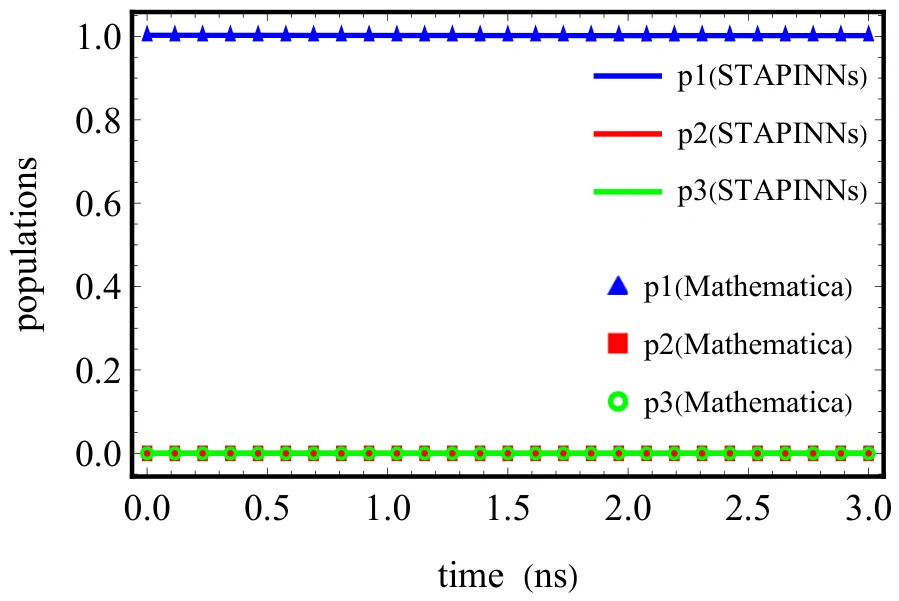
The population transfer simulated by the STAPINNs method without driving. The green curve represents the population of state |1〉, the blue curve that of state |3〉, and the red curve that of the intermediate state |2〉. During training, parameter values are taken as $\Omega_{0}=0.01\pi \;\mathrm{GHz},\;t_{f}=3\;\mathrm{ns},$ $\sigma =t_{f}/6$, $\tau =t_{f}/10$, Δ=4πGHz, Γ=0.1πGHz, η=0.005, epoch=7000, N=50.

We first simulate population transfer without an additional driving term, as shown in Figure 2. In the presence of dissipation, with parameters chosen as in Figure 2, the neural network is trained by adjusting its parameters through the loss function defined above. When the loss function converges, the neural network is used to fit the evolution process, yielding the population transfer curve. Next, the same differential equation is solved using Mathematica 11 to obtain the population transfer curve. A comparison of the curves obtained by the two methods is shown in Figure 2. Among them, η is the learning rate, epoch is the number of training epochs, and N is the number of samples. The figure shows that, in the absence of additional driving, the population transfer curves produced by the PINN method and by Mathematica are consistent. This demonstrates that using STAPINNs for numerical simulation of three-level quantum system evolution yields correct and accurate results.

The driving function $\Omega_{a}\left(t\right)$ can be regarded as a time-dependent parameter of the differential equation. The parameterized differential equation is incorporated as a constraint into the loss function of the neural network to train the network. During training, as the number of training epochs increases, the error of the loss function associated with the differential equation decreases.

**Figure 3:**
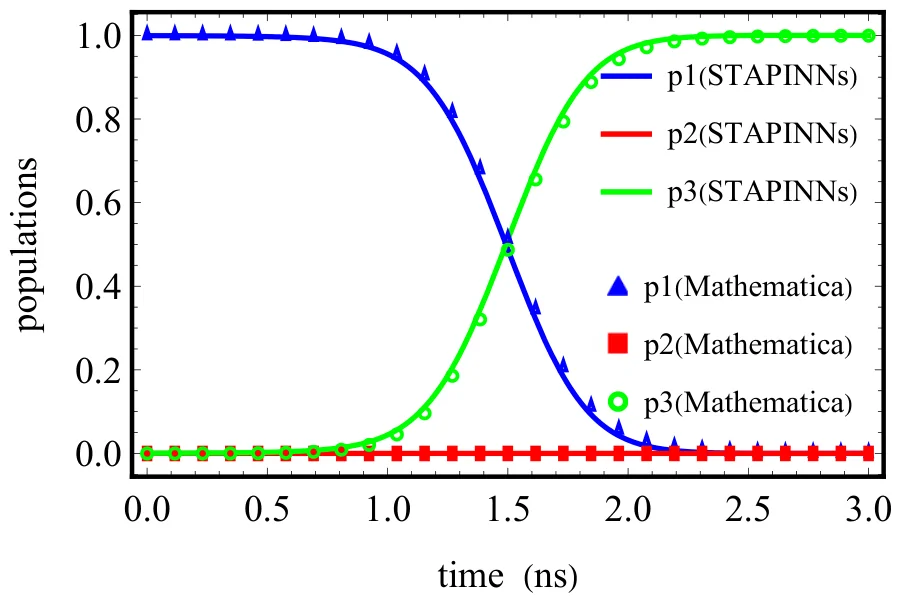
Population inversion achieved by the STAPINNs method. The green curve represents the population of state |1〉, the blue curve that of state |3〉, and the red curve that of the intermediate state |2〉. The solid lines are the results fitted by STAPINNs, and the dashed lines are the simulation results obtained by solving the system evolution with Mathematica. During training, parameter values are taken as $\Omega_{0}=0.01\pi \;\mathrm{GHz}$, $t_{f}=3\;\mathrm{ns}$, $\sigma =t_{f}/6$, $\tau =t_{f}/10$, Δ=4πGHz, Γ=0.1πGHz, epoch=7000, η=0.005, N=50.

The trained STAPINNs can then fit the system evolution process. Figure 3 shows the population transfer curves after training. It can be observed that population inversion is achieved at time t=2.5ns, and the system remains in a stable population inversion state until the final time t=3ns of the evolution. While approximating the adiabatic evolution process, the trained neural network also yields a driving function that satisfies the population inversion condition. Using the same parameters, the obtained driving function is substituted into the differential equation to eliminate the time-dependent parameters. The system evolution is then simulated using Mathematica, producing the evolution curves shown in Figure 3. A comparison shows that the results from STAPINNs are consistent with those from Mathematica, confirming the correctness and effectiveness of the STAPINNs method. From the evolution curves in Figure 3, it can be seen that the curves are antisymmetric about the midpoint t=1.5ns of the evolution time. Specifically, the first half of the population curve for state |1〉 is symmetric with the second half of the population curve for state |3〉 about t=1.5ns.

**Figure 4:**
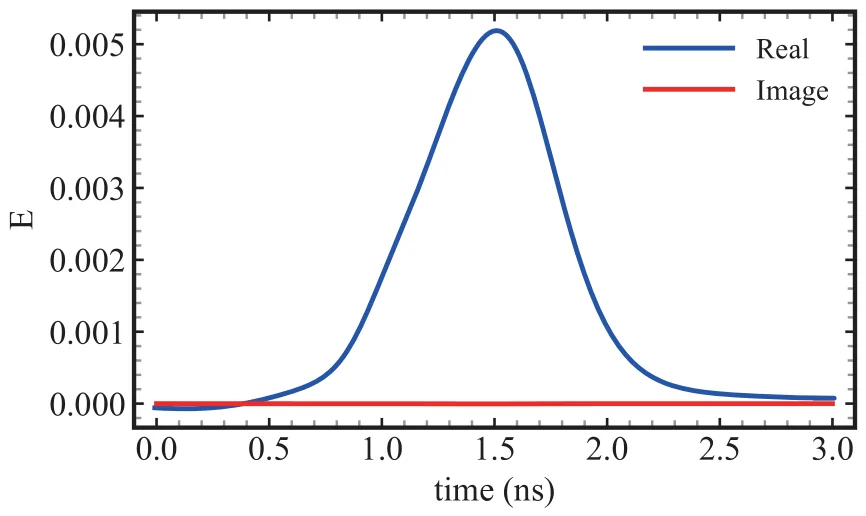
Time evolution of the system expectation. The red curve denotes the imaginary part of the Hamiltonian expectation value, and the blue curve denotes its real part. During training, the parameters are set as follows: $\Omega_{0}=0.01\pi \;\mathrm{GHz}$, $t_{f}=3\;\mathrm{ns}$, $\sigma =t_{f}/6$, $\tau =t_{f}/10$, Δ=4πGHz, Γ=0.1πGHz, epoch=7000, η=0.005, N=50.

Using the parameters in Figure 4, the neural network is trained, and the results are obtained. The system expectation values are then calculated. The curves of the expectation values as functions of time are shown below. The figure shows that the imaginary part of the expectation value is a straight line identically equal to zero within numerical accuracy, indicating that the expectation value is real along the constrained trajectory. The real part curves in the figure are symmetric about t=1.5ns.

**Figure 5:**
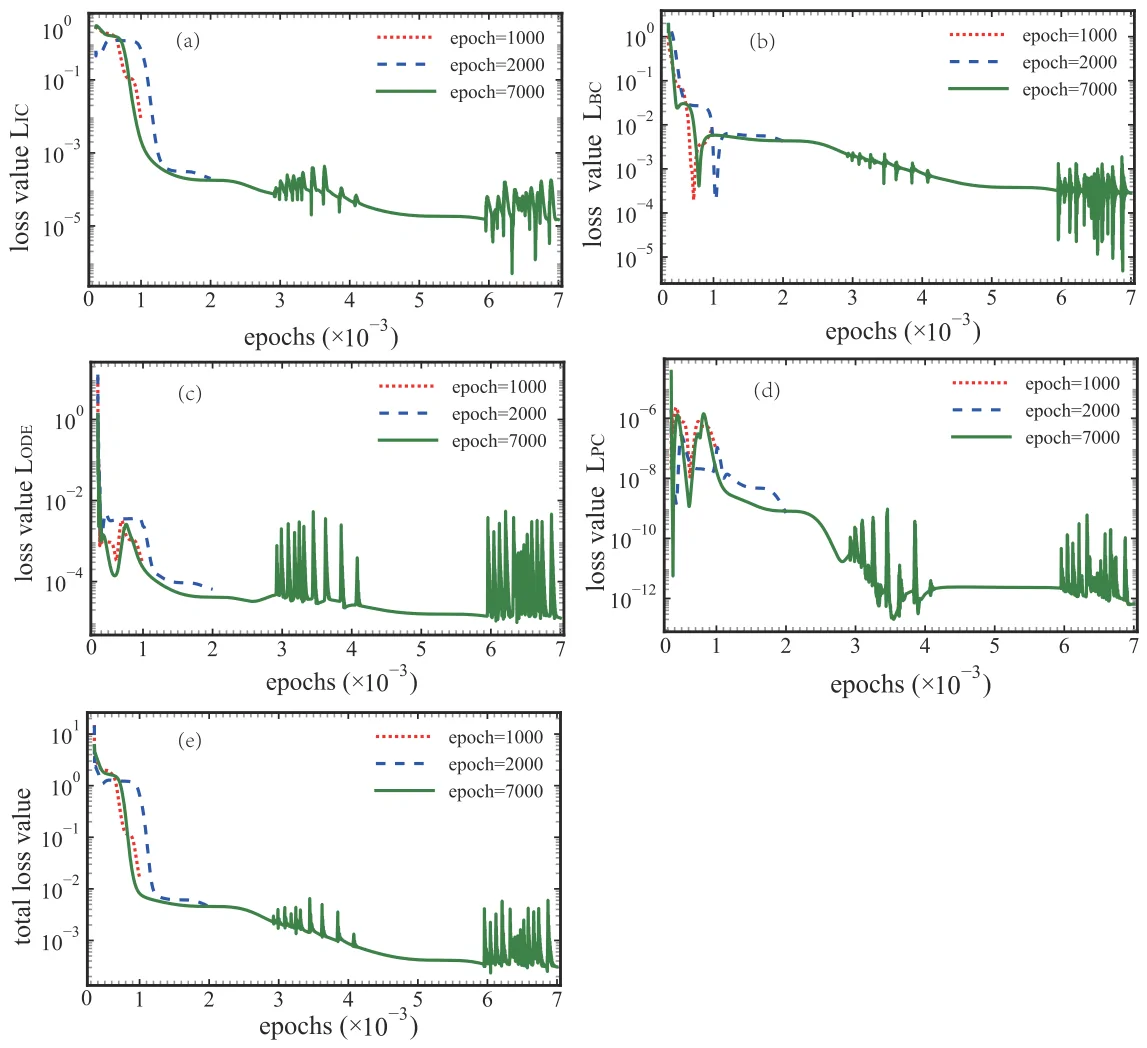
Training dynamics of the STAPINNs loss function. Panels (**a**–**d**) show the individual loss components: (**a**) initial condition loss $L_{\mathrm{IC}}$, (**b**) target population and boundary loss $L_{\mathrm{BC}}$, (**c**) dynamical residual loss $L_{\mathrm{ODE}}$, and (**d**) instantaneous norm constraint loss $L_{\mathrm{PC}}$. Panel (**e**) displays the total loss $L_{\mathrm{OSS}}$, and panel. The training was performed with epochs of 1000 (red dashed), 2000 (blue dashed), and 7000 (green solid), respectively. The physical parameters are $\Omega_{0}=0.01\pi \;\mathrm{GHz}$, $t_{f}=3\;\mathrm{ns}$, $\sigma =t_{f}/6$, $\tau =t_{f}/10$, Δ=4πGHz, Γ=0.1πGHz, η=0.005, and N=50.

**Figure 6:**
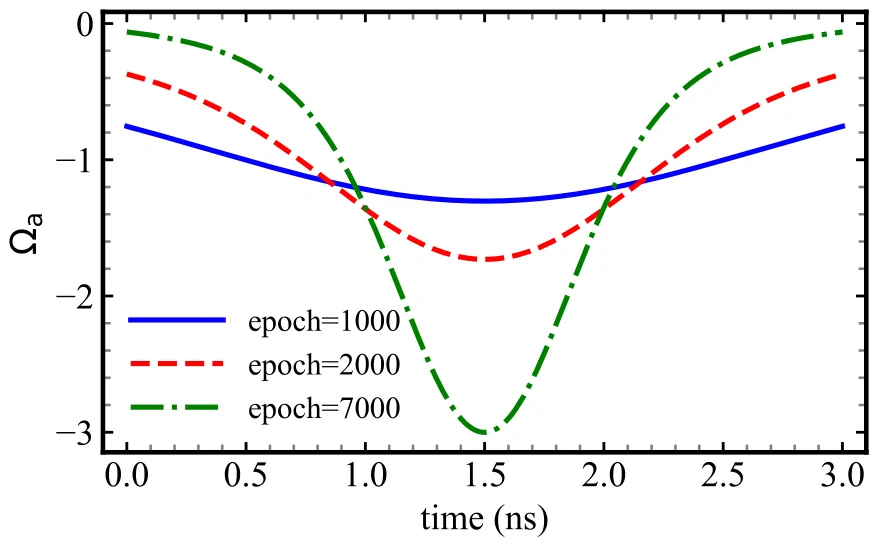
Optimized driving functions obtained for training epochs of epoch=1000, 2000, and 7000, respectively. The solid blue curve corresponds to 1000 epochs, the dashed red curve to 2000 epochs, and the dashed green curve to 7000 epochs. The parameters are set as $\Omega_{0}=0.01\pi \;\mathrm{GHz}$, $t_{f}=3\;\mathrm{ns}$, $\sigma =t_{f}/6$, $\tau =t_{f}/10$, Δ=4πGHz, Γ=0.1πGHz, η=0.005, N=50.

**Figure 7:**
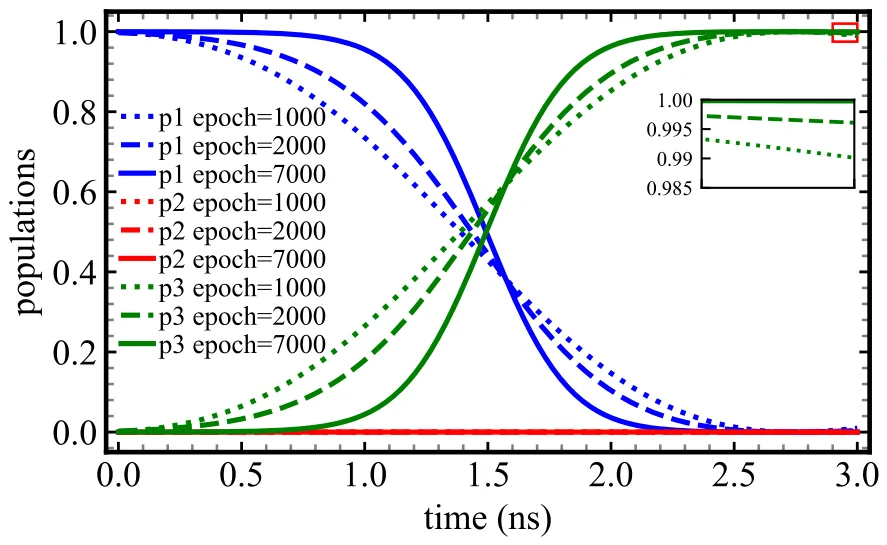
Population transfer curves for different numbers of training epochs. The three green curves represent the population of state |1〉 for different epochs, the three blue curves represent the population of state |3〉, and the three red curves represent the intermediate state |2〉. The parameters are set as follows: $\Omega_{0}=0.01\pi \;\mathrm{GHz},t_{f}=3\;\mathrm{ns}$, $\sigma =t_{f}/6$, $\tau =t_{f}/10$, Δ=4πGHz,Γ=0.1πGHz,η=0.005, N=50, epoch=7000.

Next, the neural network is trained with different numbers of training epochs. To examine the training behavior of the STAPINNs framework, we plot in Figure 5 the evolution of the individual loss components and the total loss over training epochs. Figure 5a–d show the initial condition loss $L_{\mathrm{IC}}$, the dynamical residual loss $L_{\mathrm{ODE}}$, the boundary loss $L_{\mathrm{BC}}$, and the probability conservation loss $L_{\mathrm{PC}}$, respectively. All four components decrease steadily as training proceeds, indicating that the network progressively satisfies the physical constraints encoded in the loss function. Figure 5e presents the total loss $L_{\mathrm{OSS}}$, which exhibits a consistent downward trend and eventually falls below $10^{-3}$ after approximately 7000 epochs. The corresponding optimized driving fields and population transfer curves obtained from these three epoch settings are shown in Figure 6 and Figure 7, respectively.

Figure 6 shows the driving function curves obtained at different training epochs. The results show that when the number of epochs reaches 7000, the curve becomes symmetric with respect to time t=1.5,ns. Figure 7 compares the time-dependent population curves for epoch = 1000, 2000, 7000; as mentioned earlier, they also exhibit clear time-reversal symmetry between states |1〉 and |3〉.

**Figure 8:**
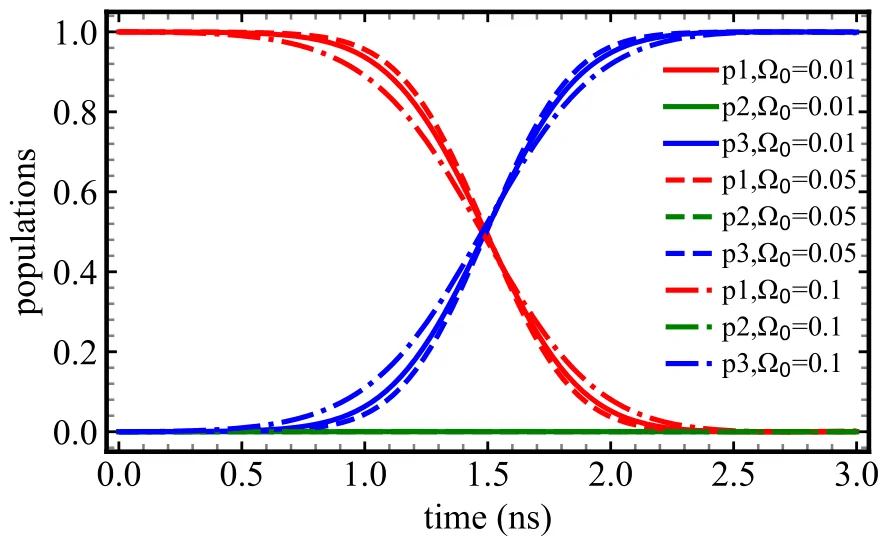
Population transfer curves for different values of $\Omega_{0}$. The parameters are set as follows: $\Omega_{0}=0.01\pi ,\;0.05\pi ,\;0.1\pi \;\mathrm{GHz}$, $t_{f}=3\;\mathrm{ns}$, $\sigma =t_{f}/6$, $\tau =t_{f}/10$, Δ=4πGHz, Γ=0.1πGHz, η=0.005, N=50, and training epochs epoch=7000. The solid curve corresponds to $\Omega_{0}=0.01\pi \;\mathrm{GHz}$, the dashed curve to $\Omega_{0}=0.05\pi \;\mathrm{GHz}$, and the dash-dot curve to $\Omega_{0}=0.1\pi \;\mathrm{GHz}$.

A different set of parameters is chosen for comparison, with $\Omega_{0}=\left[0.01,0.05,0.1\right]\pi \;\mathrm{GHz}$. The remaining parameters are as shown in Figure 8. To evaluate the generalization capability of the STAPINNs framework with respect to the coupling strength, we retrain the network independently for each chosen value of $\Omega_{0}$ and examine whether a high-fidelity control pulse can still be obtained. The results, shown in Figure 8, demonstrate that the method successfully adapts to a broad range of $\Omega_{0}$, yielding fidelities exceeding 0.999 in all cases. This indicates that the proposed inverse design framework can consistently produce ideal solutions without fine-tuning of the coupling strength parameter.

The driving function $\Omega_{a}\left(t\right)$ obtained by STAPINNs is a discrete-valued function. In this work, the discrete driving function obtained from the neural network is expanded using both Fourier series and polynomial methods. Specifically, $\Omega_{a}\left(t\right)$ is expanded into a Fourier series to generate a pulse function composed of elementary trigonometric functions. The Fourier series formula is given as follows:

(21) $$\Omega_{a}\left(t\right)=\frac{a_{0}}{2}+\sum_{n=1}^{\infty }\left[a_{n}\cos\left(\frac{2\pi nt}{T}\right)+b_{n}\sin\left(\frac{2\pi nt}{T}\right)\right],$$

where the Fourier coefficients are

(22) $$a_{n}=\frac{2}{T}\int_{t_{0}}^{t_{0}+T}\Omega_{a}\left(t\right)\cdot \cos\left(\frac{2\pi nt}{T}\right)dt,\;n=0,1,2,\ldots$$

(23) $$b_{n}=\frac{2}{T}\int_{t_{0}}^{t_{0}+T}\Omega_{a}\left(t\right)\cdot \sin\left(\frac{2\pi nt}{T}\right)dt,\;n=0,1,2,\ldots \;$$

The calculated Fourier coefficients are

(24) $$\begin{array}{cc} a_{n}=(-1.5583\times 10^{0},\; & 9.6313\times 10^{-1},-5.4213\times 10^{-1},2.7820\times 10^{-1},-1.2869\times 10^{-1},\\ 5.6308\times 10^{-2},\; & -1.8474\times 10^{-2},7.4633\times 10^{-3},1.2269\times 10^{-3},1.1456\times 10^{-3},\\ 2.7087\times 10^{-3},\; & 1.4249\times 10^{-3},2.2730\times 10^{-3},1.8540\times 10^{-3},2.0753\times 10^{-3},\\ 1.9993\times 10^{-3},\; & 2.0430\times 10^{-3},2.0382\times 10^{-3},2.0487\times 10^{-3},2.0516\times 10^{-3},\\ 2.0561\times 10^{-3},\; & 2.0583\times 10^{-3},2.0603\times 10^{-3},2.0608\times 10^{-3}); \end{array}$$

(25) $$\begin{array}{cc} b_{n}=(7.9858\times 10^{-3},\; & 2.4058\times 10^{-2},1.0632\times 10^{-3},8.8094\times 10^{-4},1.1331\times 10^{-2},\\ -6.2202\times 10^{-3},\; & 1.0510\times 10^{-2},-4.3276\times 10^{-3},6.2125\times 10^{-3},-1.3457\times 10^{-3},\\ 3.0515\times 10^{-3},\; & 2.2882\times 10^{-4},1.5210\times 10^{-3},6.7984\times 10^{-4},8.9194\times 10^{-4},\\ 6.5546\times 10^{-4},\; & 6.0389\times 10^{-4},5.0378\times 10^{-4},4.1771\times 10^{-4},3.3959\times 10^{-4},\\ 2.5919\times 10^{-4},\; & 1.8452\times 10^{-4},1.0976\times 10^{-4},3.6503\times 10^{-5}); \end{array}$$

(26) $$a_{0}=\frac{2.0345}{2}.$$

The formula for the polynomial expansion is as follows:

(27) $$\Omega_{a}\left(t\right)=\sum_{n=0}^{18}\lambda_{n}t^{n}.$$

The coefficients are

(28) $$\begin{array}{cc} \lambda_{n}=(-5.5658\times 10^{2},\; & 3.2443\times 10^{3},2.4129\times 10^{3},-1.3928\times 10^{4},-4.3334\times 10^{3},\\ 2.4842\times 10^{4},\; & 4.1626\times 10^{3},-2.3855\times 10^{4},-2.3003\times 10^{3},1.3376\times 10^{4},\\ 7.2900\times 10^{2},\; & -4.4506\times 10^{3},-1.2291\times 10^{2},8.5800\times 10^{2},8.7633\times 10^{0},\\ -9.0243\times 10^{1},\; & -9.7297\times 10^{-2},4.5806\times 10^{0}); \end{array}$$

**Figure 9:**
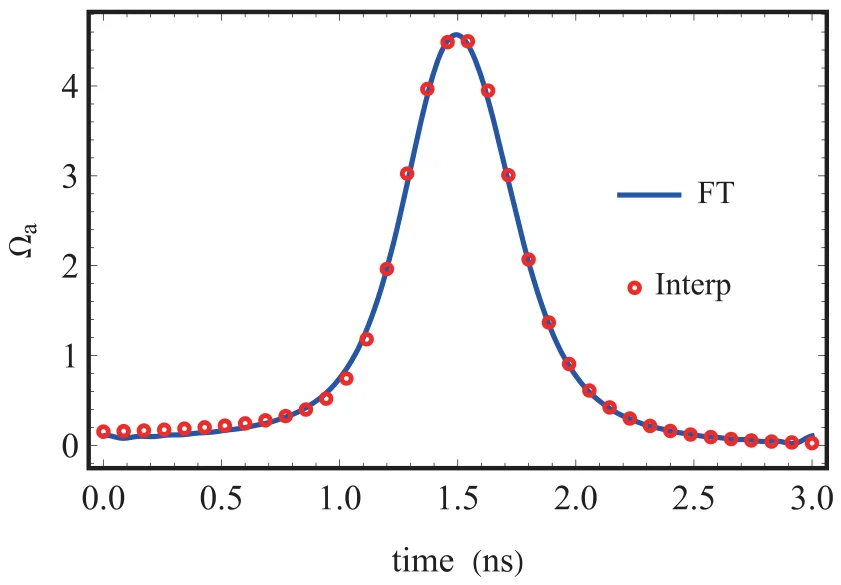
Comparison of the driving functions obtained by polynomial expansion and Fourier series expansion. The red dashed curve represents the polynomial expansion of the driving function obtained by STAPINNs, and the blue solid curve represents the Fourier series expansion of the driving function obtained using Mathematica. The parameters are set as $\Omega_{0}=0.1\pi \;\mathrm{GHz}$, $t_{f}=3\;\mathrm{ns}$, $\sigma =t_{f}/6$, $\tau =t_{f}/10$, Δ=4πGHz, Γ=2πGHz, epoch=7000, η=0.005, N=50.

**Figure 10:**
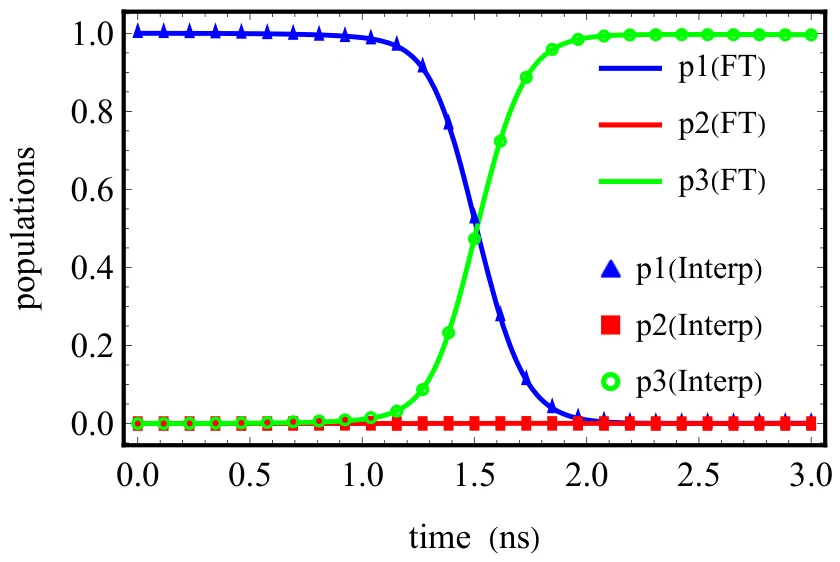
Comparison of population transfer curves obtained using the driving functions from polynomial expansion and Fourier series expansion. The solid curves (various colors) represent the population transfer solved by substituting the Fourier-transformed driving function into the differential equation and solving it with Mathematica. The dashed curves (corresponding colors) represent the population transfer obtained using the polynomial-expanded driving function. The parameters are $\Omega_{0}=0.1\pi \;\mathrm{GHz}$, $t_{f}=3\;\mathrm{ns}$, $\sigma =t_{f}/6$, $\tau =t_{f}/10$, Δ=4πGHz, Γ=2πGHz, η=0.005, N=50, epoch=7000.

The two curves in Figure 9 represent the analytical approximations of the driving function obtained by Fourier series and polynomial interpolation, respectively. Substituting the two expanded analytical functions $\Omega_{a}\left(t\right)$ into the original differential equation transforms it from a parameterized equation into a deterministic one. This equation is then solved using Mathematica, and the resulting evolution is shown in Figure 10. The figure shows that fitting the numerical function to an analytical form using either the Fourier series or polynomial method introduces some deviation. Nevertheless, the overall evolution is highly consistent with that in Figure 3, and population inversion is achieved in both cases. This further verifies that the obtained driving function is correct and effective. It can also be observed from Figure 9 that the driving function is symmetric about t=1.5ns.

**Figure 11:**
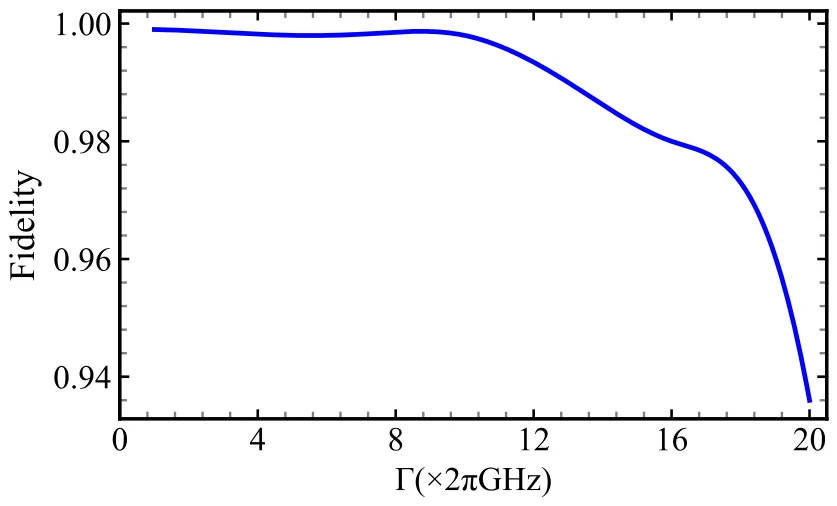
Fidelity as a function of the dissipation coefficient. The curve shows how fidelity varies with Γ. The parameters are set as $\Omega_{0}=0.1\pi \;\mathrm{GHz}$, $t_{f}=3\;\mathrm{ns}$, $\sigma =t_{f}/6$, $\tau =t_{f}/10$, Δ=4πGHz, and Γ ranges from 2πGHz to 40πGHz. η=0.005, N=50, epoch=7000.

To assess the adaptability of the STAPINNs method to varying dissipation rates, we define the fidelity as $F=|c_{3}\left(t_{f}\right){|}^{2}$, i.e., the final population of the target state |3〉. We then retrain the network from scratch for each distinct value of Γ while keeping all other physical parameters fixed. As shown in Figure 11 the fidelity exhibits a gradual decline as Γ increases yet remains above 0.99 when Γ is increased by a factor of 10, and above 0.93 even for a 20-fold increase. This demonstrates that the STAPINNs framework exhibits strong generalization capability across a wide range of dissipation coefficients, consistently yielding high-fidelity control pulses without requiring parameter-specific adjustments to the network architecture or training strategy.

Regarding the experimental implementation of the learned control pulse, we note that the numerical function $\Omega_{a}\left(t\right)$ obtained by STAPINNs can be conveniently represented by a Fourier series, as shown in Equation (21). This expansion directly provides a superposition of harmonic components, which can be readily generated using an arbitrary waveform generator (AWG) or a phase-modulated laser/microwave field in typical quantum optics and superconducting circuit experiments. The dominant low-frequency Fourier coefficients determine the overall pulse envelope, while the high-frequency components offer fine-tuning capability. In practice, additional constraints such as bandwidth limitations and maximum amplitude of the experimental apparatus may affect the pulse shape. However, our analysis of the generalization capability over a range of $\Omega_{0}$ and Γ values indicates that the scheme maintains high fidelity under various physical conditions. Furthermore, the Fourier representation allows for a straightforward truncation of high-frequency terms, which facilitates the implementation without significant performance degradation. This provides a practical pathway from the numerically learned pulse to an experimentally feasible control field.

## 4. Conclusions

In this paper, we proposed a physics-informed neural network scheme for inverse design of shortcuts to adiabaticity in a three-level non-Hermitian quantum system. The PINN simultaneously learns the state amplitudes and the auxiliary driving field by minimizing the Schrödinger residual together with the initial condition loss, the boundary population loss and the probability conservation loss. The corrected dynamical equations include the dissipative contribution, ensuring consistency between the Hamiltonian, the PINN training objective, and independent propagation verification. Numerical experiments demonstrate that the learned compensation field can drive high-fidelity population inversion. To evaluate the generalization capability of the proposed framework, we retrain the network independently for each chosen value of the physical parameters. The results show that the method consistently yields high-fidelity control pulses over the tested ranges of coupling strength $\Omega_{0}$ and dissipation coefficient Γ, confirming its adaptability across different parameter regimes. This procedure demonstrates the flexibility of the inverse design framework when applied to new parameter settings, rather than the robustness of a particular fixed pulse shape. The imposed probability conservation constraint yields a real Hamiltonian expectation value along the learned trajectory within numerical accuracy, although we do not claim that this trajectory-level result establishes pseudo-Hermiticity of the full Hamiltonian or real expectation values for all observables. The observed approximate time-reversal symmetry in the population dynamics follows from the chosen pulse symmetry and the Hamiltonian structure. These results indicate that PINNs provide a flexible and effective constrained inverse design tool for non-Hermitian STA protocols. Future work will include systematic comparisons with conventional STA and optimal control methods, as well as more detailed convergence analyses, and will extend the present approach to multi-level and many-body open quantum systems. 

## Data Availability

Data are contained within the article.
